# An exploration of automated narrative analysis via machine learning

**DOI:** 10.1371/journal.pone.0224634

**Published:** 2019-10-31

**Authors:** Sharad Jones, Carly Fox, Sandra Gillam, Ronald B. Gillam

**Affiliations:** 1 Department of Mathematics and Statistics, Utah State University, Logan, Utah, United States of America; 2 Department of Special Education and Rehabilitation, Utah State University, Logan, Utah, United States of America; 3 Department of Communication Disorders and Deaf Education, Utah State University, Logan, Utah, United States of America; University of Aveiro, NEW ZEALAND

## Abstract

The accuracy of four machine learning methods in predicting narrative macrostructure scores was compared to scores obtained by human raters utilizing a criterion-referenced progress monitoring rubric. The machine learning methods that were explored covered methods that utilized hand-engineered features, as well as those that learn directly from the raw text. The predictive models were trained on a corpus of 414 narratives from a normative sample of school-aged children (5;0-9;11) who were given a standardized measure of narrative proficiency. Performance was measured using Quadratic Weighted Kappa, a metric of inter-rater reliability. The results indicated that one model, BERT, not only achieved significantly higher scoring accuracy than the other methods, but was consistent with scores obtained by human raters using a valid and reliable rubric. The findings from this study suggest that a machine learning method, specifically, BERT, shows promise as a way to automate the scoring of narrative macrostructure for potential use in clinical practice.

## Introduction

There has long been a need to have cost effective, efficient and reliable means for evaluating student writing and language abilities. Automatic essay scoring (AES) technology was first introduced by Ellis Page in 1966 when he developed Project Essay Grader, a software that produces computer-generated scores based on writing features such as grammaticality, essay length and organization (PEG) [1–3]. AES systems, such as PEG, are developed to increase the speed and ease of essay scoring, with the intention of either replacing or supplementing human scorers [1, 4–6]. Such systems operate on predictive models, which analyze text and output standardized scores at varying levels of predictive accuracy, as measured by their correlation with human-produced scores [1]. Many other AES systems have been developed since the introduction of PEG to increase both the accuracy of predictive models, as well as to offer additional features, such as plagiarism checks and critical feedback [1, 7].

There are currently two main domains of AES systems: those that utilize hand-engineered essay features and those that utilize raw-text approaches [1, 7]. Hand-engineered features have long been the standard approach as they use knowledge provided by a domain expert to create numeric representations of the text, allowing for simple statistical modeling techniques to identify relevant patterns for scoring. Raw text approaches use statistical models, often neural networks, to map the space of possible words to a higher dimensional embedding space, which typically encodes a more semantic understanding of the word. Using these vectors, machine learning (ML) methods can be applied, either to the sequence of text in order or to the “bag-of-words”, to construct a model for scoring the text. [7]

### Limitations of AES in clinical scenarios

While there are seemingly a large number of applications of AES technologies, these systems have primarily been developed for the purposes of scoring high-stakes written- assessments, such as the SAT and GRE [1]. Well-known systems such as PEG, E-Rater and IntelliMetric are proprietary technologies that are not open-sourced to the public, so while AES technology is very useful, it is often not readily accessible. There is therefore a need for the development of open-access AES technology if it is to be useful for clinical practitioners in various fields such as psychology, speech language pathology or education.

An additional drawback of many AES systems is that they operate on holistic scoring methods, which attempt to judge the overall writing quality based on a 5-6 point scale [1]. While this is the chosen design for scoring standardized tests like the GRE, rubric-based scoring systems can give more nuanced feedback by providing input on particular areas of strength or weakness in an individual’s writing [8]. Rubric-based tools are often used by educators, psychologists and clinicians, to assess changes in children’s language abilities overtime, as well as indicate areas of language deficits [9]. In particular, clinicians such as Speech Language Pathologists (SLPs) are encouraged to collect language samples from their clients to diagnose areas of strengths and weaknesses, and to monitor progress over the course of intervention. Narrative language samples are the preferred elicitation context during the school-age years, however, many clinicians do not use them because of time-constraints. [10, 11].

### Narrative sample analysis

One benefit of narratives, or stories, are that they are a universal form of discourse, and are not as subject to the cultural biases commonly seen in standardized assessment. Research on narrative discourse shows that the same macrostructural elements, also referred to as “story grammar” [12, 13], are consistent across many cultures [14, 15]. As is outlined in Stein and Glenn [16], the basic elements of an episode include the initiating event, action and consequence, which are linked by both causal and temporal connections (e.g. A bear appeared, *and then* she ran way *so that* she would not be eaten). Stories are comprised of settings plus episodes. Narrators can include other story elements into the macrostructure such as internal responses, plans and reactions to elaborate on episodes. Prior research has shown that children with delayed or impaired language abilities include these macrostructure elements less frequently, and in less elaborated forms than their typically developing peers [17–19], making narrative sampling an informative assessment tool. Narrative evaluation and progress-monitoring tools, such as the Test of Narrative Language [20] and the Monitoring Indicators of Scholarly Language (MISL) [9] provide standardized and criterion-referenced assessments of macrostructural elements that allow clinicians to quantify the quality of a child’s narrative to make diagnostic and treatment decisions.

#### Barriers to narrative sampling

In an ideal world, teachers and SLPs would use narrative sampling and progress-monitoring tools on a regular basis. Unfortunately this is often not the case. In a survey of 1,399 SLPs, the most frequently reported barrier to the use of non-standardized assessment tools, such as narrative language sampling, was time [10]. Most teachers and SLP’s work with large numbers of students, and operate under both time and resource constraints. While narrative progress-monitoring tools are a valid source of information about changes in a child’s language abilities after instruction, they are time-consuming to elicit and score, and often require extensive training [9]. These barriers lessen the practicality of such tools, making teachers and SLPs alike, less likely to use them. Though AES technology exists, limited effort has been made to automate clinical tools for use in clinical settings.

### Existing technology & gaps in the literature

Two of the most commonly used language analysis tools are the Systematic Analysis of Language Transcripts (SALT) [21] and the Child Language Data Exchange System’s (CHILDES) program CLAN [22]. These are both software programs that allow educators, clinicians and psychologists to transcribe language samples while inserting various codes to obtain clinically useful information about aspects of language including mean length of utterance, total number of words, total number of different words, grammaticality, syntactic complexity and narrative quality. Unfortunately, teachers and SLP’s report that even when required to obtain and use language sampling techniques in their practices, they often do not do so because while valuable, these methods take more time to complete than they have available [10].

Coh-Metrix is a text-analysis tool that is open-access, and provides over one-hundred distinct measures of language features [23]. Though this is a useful tool for many contexts, it provides only a handful of measures that are directed towards narrative analysis, with a greater focus on text cohesion [24]. In 2018, researchers working under ETS came close to creating a clinically relevant automated narrative sample analysis system [25]. This work serves as an important proof of concept, however it still leaves a number of gaps in the literature. For one, while the system is rubric-based, it was teacher-developed and addressed only a few key macrostructural components important for the development of complete, coherent and quality narratives. In addition, their system was was not associated with good scoring reliability perhaps due in part of their use of somewhat simple statistical techniques (i.e. linear regression). This system was designed as an alternative to E-Rater, an AES system developed by ETS for scoring narrative essays, and while it may be suitable to that purpose, it likely does not translate to clinical assessment.

### The current study

Given the limited technology available to clinicians and SLPs for completing automated scoring of narrative language samples, we sought to investigate the feasibility of designing such a system. In this study, we aimed to explore various methodologies in the development of a computer-based narrative analysis system to automate the scoring of aspects of narrative macrostructure to include the following elements: character, setting, initiating event, plan, action, consequence and elaborated noun phrase. The current study was designed to answer whether an automated narrative scoring system could generate scores at or above a reliability levels achieved by human-raters.

## Materials and methods

### Ethics statement

This study was conducted under full written institutional review board (IRB) approval by the Utah State University IRB under protocol # 9802 and approval number FWA#00003308, which was assessed as minimal risk.

The approval statement is as follows: Your proposal has been reviewed by the Institutional Review Board and is approved under expedite procedure #6 (based on the Department of Health and Human Services (DHHS) regulations for the protection of human research subjects, 45 CFR Part 46, as amended to include provisions of the Federal Policy for the Protection of Human Subjects, November 9, 1998): Collection of data from voice, video, digital, or image recordings made for research purposes.

### Corpus

The corpus included in this study consisted of 414 oral narratives from a normative sample of school-aged children (5;0-9;11). Narratives were elicited in response to the *Alien Story* prompt from the Test of Narrative Language-2, which is single-scene used to elicit a story [20]. Narratives produced by the children were digitally recorded and transcribed, using Systematic Analysis of Language Transcripts, based on the conventions outlined in Miller & Chapman [21]. Transcription was completed verbatim by a team of trained research assistants, all of whom were blinded to the purpose of the study. Reliability between transcribers was determined by examining 20% of the transcripts and was calculated as percentage agreement for both C-Units (i.e. number of independent clauses and their attached dependent clauses within a narrative) and mazing (i.e. segments of transcript that were excluded from analysis), averaging at 96%.

### Narrative macrostructure hand-scores

The MISL is a rubric-based progress-monitoring tool designed to assess the quality of school-aged children’s narratives, based on both macrostructural (i.e. story grammar) and microstructural (i.e. sentence level) elements [9]. For the purposes of this study, primarily macrostructural elements were included. Each of the transcripts within the corpus had been previously hand-scored with the MISL rubric. MISL hand-scores for all 414 transcripts were completed by a group of trained undergraduates scorers, whom had previously reached or surpassed an inter-rater reliability level of 85%. An additional 50 transcripts were randomly selected and double-scored by an expert scorer; a doctoral student with more than three years of MISL scoring experience. Each narrative within the corpus was given a MISL overall macrostructure score that was calculated by adding each individual element.

There are a total of six macrostructure elements contained in the MISL that are discussed in this study, including character, setting, initiating event, plan, action and consequence. We also included one element from the microstructure section of the MISL, elaborated noun phrase (ENP). ENP is a measure of the number of modifiers that precede a noun, such as in the sentence *the large yellow* **house**. While ENP does not fall under macrostructure, it was determined to be less suited to hard-coded automation than the other microstructure elements on the MISL (i.e. subordinating conjunctions, adverbs, etc.). ENP requires both the classification of individual words within a narrative (i.e. verb, noun, adjective) and identifying the proper sequence of words prior to a noun (i.e. article + adjective + noun, etc.). In a separate study which evaluated the hard-coded automation of microstructure scoring, ENP had the lowest accuracy level. ENP was therefore included in this study in an attempt to improve its automated scoring accuracy.

Full definitions of all elements can be found below in Table 1. Each element is scored on a scale of 0 to 3, where 0 indicates that the element is not present and 3 indicates that it has been mastered. The elements of character and setting are scored based on their degree of elaboration, whereby naming the main character(s)and describing both the specific place and time of the setting, will result in a higher score than simply stating: *There was a boy in a house. The scores for* the remaining elements are dependent on the initiating event. If a child does not include an initiating event (receiving a score of 0 or 1), then the maximum score for plan, action and consequence is 1. Further, in order to score a 2 or higher on each element, they must state an explicit causal link between the initiating event and the actions and consequences that follow. This was an important consideration in the development of our predictive models, because low QWK in the prediction of initiating event would likely result in low performance on nearly all other measures.

**Table 1:** Definition of MISL macrostructure elements and ENP.

MISL Element	Definition
*Character*	The who or what in the story acting as the agent
*Setting*	The time and/or place the story or episode takes place
*Initiating Event*	An event or problem that causes the story to “take-off”
*Plan*	The idea the character(s) has to fix the problem in the story
*Action*	The action taken by the character in response to the initiating event
*Consequence*	A causally linked event following the character’s action
*Elaborated Noun Phrase*	The number of modifiers following a given noun, that serve to describe the noun

For full definitions of macrostructure elements reference [9].

### Data cleaning

Prior to analysis, the corpus was read into R, an open-source statistical computing software, where all unwanted characters were removed or “cleaned”. The corpus had been previously transcribed in SALT software and thus contained a large number of unwanted characters, including backslashes, asterisks, parentheses and time stamps. Rules for determining the characters to remove were hard-coded in R using “if-then” logic (i.e. if “/” then replace with “”) that was written into a string manipulation function. The function operated by analyzing individual transcripts as a string of text, searching for the designated unwanted characters, and finally replacing them line by line with either a new character or blank space. The function was further written into a for-loop, allowing the entire corpus to be automatically cleaned within one execution of the code. The code for this function can be found doi: https://OSF.IO/BCZPQ under *app.R*. Cleaned transcripts were then combined into one data-set, with each transcript tagged with an unique identifier matching its corresponding MISL scores.

### Modeling methods

Given that our data consist of labeled observations (i.e. each narrative has associated known scores), there existed a large number of possible supervised learning techniques in machine learning which we could explore. To constrain the search of possible methods, we chose four methods that not only span the range of complexity in Natural Language Processing (NLP), but also take distinctly different approaches to quantifying text data. Two of these methods use hand-engineered features to preprocess the data, Coh-Metrix with Random Forests (CMRF) and TF-IDF with Random Forest (TIRF), and two directly handle the raw text, GloVe Embeddings with LSTM’s (GVEL) and Bidirectional Encoder Representations from Transformers (BERT). Each method proposes a different approach to the same end, predicting the macrostructure MISL scores, and also follows a common pattern: Take as input the raw text or pre-processed narratives, train a model that maps inputs to outputs (i.e. MISL scores) using a ML algorithm, and then use the trained model to predict outputs on unseen data.

#### Coh-Metrix with Random Forests (CMRF) & TF-IDF with Random Forests (TIRF)

The first two approaches explored, CMRF and TIRF, provide a simple and easily reproduced baseline through the utilization of Random Forests (RF) [26]. While there are many other viable choices of learning algorithms (e.g. Linear Regression, Support Vector Machines, Gradient Boosting Machines, etc.), RF’s were specifically selected due to their strength and flexibility as a learning algorithm, while being very robust to hyperparameter selections. This minimizes the possibility of overfitting our solution given that we have a relatively small data set. RF’s take as input a numeric matrix consisting of rows, representing narratives, and columns, representing variables. The two methods vary in the way they generate the variables in this matrix.

Coh-Metrix is a tool developed to quantify the cohesion and coherence across many different metrics, through dimensionality reduction techniques (e.g. PCA and LSA) and the identification of specific syntactical structures, resulting in 108 unique measures or variables [23]. TF-IDF takes a simpler approach, in that the variables of the matrix represent all unique words across the corpus of narratives and each row is an individual narrative. The entries of this matrix are a calculation of the frequency of a given word in a single narrative divided by the frequency of that word across all narratives. This has the effect of down-weighting common words in a given set of texts, while upweighting words in a text that may not be used as often. Conceptually, this should allow a machine learning method to identify sets of words that contribute to specific scores (if any pattern exists).

While the RF based methods provide a strong baseline, most modern NLP methods employ some form of a neural network. The main motivation for this, is the need to consider the ordering and context of words in a given narrative. This is especially true when trying to automatically predict macrostructure elements, which often contain long term or contextual dependencies. Also, RF’s are only provided with some abstracted form of the narratives from our corpus and must use the 400+ narratives to identify the differences in a given macrostructure element, with no prior knowledge of the English language. To this end, GVEL and BERT, both use pre-processing techniques that try to inject outside “knowledge” of the language and utilize neural network architectures that allow for contextual and sequential representations of words in the narratives.

#### GloVe embeddings with LSTM’s (GVEL)

GVEL pre-processes the raw text by representing each word with its Global Vectors for Word Representation (GloVe) embedding, a 300-dimensional embedding vector of numbers that is learned through pre-training on millions of Wikipedia articles [27]. This provides the model with a numeric representation (a 300 dimensional real valued vector) of each word, where many semantic relationships are encoded. A famous example of this is the vector difference between the embeddings for the words “King” and “man” is similar to the vector difference between “Queen” and “woman”. These vectors are then fed in sequentially to a Long Short-Term Memory Network (LSTM) [28], a neural network architecture that maintains a sort of “memory” state and, through the use of multiple logic gates, allows each update step to consider past actions as well as the current word vector presented. LSTMs have shown promise on many language based applications such as neural machine translation [29], video captioning [30], and text classification [31]. The success and widespread utilization of LSTMs motivates them as a viable approach to narrative scoring.

#### BERT

Bidirectional Encoder Representation from Transformer (BERT), a method developed by a team at Google in late 2018 [32], has shown state of the art results in a variety of NLP tasks including sentence pair classification, single sentence classification, question answering, and single sentence tagging, all described in detail in the original paper. A strength of BERT is that it takes this idea of learning embeddings one step further, by learning each embedding vector directly in the context of the sequence. While GloVe embeddings are learned through extensive pre-training and simply downloaded as-is for later classification tasks, BERT embeddings are learned within the process of training the specific task. This subtle but important difference, which allows homonyms and other ill-defined words to be disambiguated based on the surrounding context. BERT is still pretrained on a large corpus, however, in the process of training on our data, the whole model architecture is downloaded and modified for the specific downstream task, in our case MISL score prediction.

At its core, BERT utilizes the Transformer architecture, introduced in the paper *Attention is All You Need* by Vaswani et al. in 2017 [33]. The Transformer architecture considers an entire sequence, which we’ve defined as the entire narrative, as input and forms positionally encoded word embeddings for each word in the sequence. These embeddings are then fed into an encoding block all at once, unlike LSTM’s which take inputs sequentially. The encoding block contains a self-attention layer followed by a simple feed-forward neural network. This architecture allows each word embedding to pass through the encoding block, while acquiring relevant contextual information from surrounding words through the use of the self-attention layer. Each updated embedding is then passed on to subsequent encoding blocks before moving on to the decoder layers. Rather than performing the standard sequence-to-sequence prediction though, we instead predicted the label of a special “[CLS]” token in the output sequence. This [CLS] token represented a single score for the relevant MISL element, represented as a continuous value between 0 and 3, and was passed into the mean squared error loss function. For our final evaluations, we rounded this value to the nearest integer and calculated the quadratic weighted kappa. To summarize, we treated an entire narrative as input and predicted a single score for a given MISL element. A separate BERT model was trained for each MISL Macrostructure element.

#### Hyperparamter tuning

A key component of any ML based study is the aspect of hyperparameter tuning. In this context, hyperparameter tuning is defined as the task of choosing the parameters of the ML algorithm, which are not learned directly, but instead dictate either the architecture of the algorithm or the way in which the algorithm proceeds in learning. The difficulty of this process comes in that rigorous tuning is necessary for constructing an accurate model, but careless tuning can easily result in overfitting of the model to the training data or, in the worst case, overfitting to the test data. To avoid any information leakage in the tuning process, the data were either split into separate training and validation sets (both disjoint from the final test set) or, where it was feasible, full 10-fold cross-validation was performed on the test set for tuning the hyperparameters.

To be more specific, for Random Forests (both with Coh-Metrix and TF-IDF), effectively no tuning was done. It is well studied that Random Forests are fairly robust to their tuning parameters (number of trees and number of variables drawn at each node) and often the default parameters are sufficient for a strong tree [26]. Given that we grew these Forests in R, this meant each Forest consisted of 500 trees and, since it was a regression tree, a third of the variables were randomly selected at each node and the best split among them was taken.

For LSTM’s, an exhaustive grid search with cross-validation was carried out on the learning rate, number of layers, number of nodes, and the optimizer used. There was a bit of manual exploration performed to minimize the amount of searching required to set the bounds of the grid search effectively. The limited amount of data (≈ 400 observations) meant that the results from similar hyperparameter choices tended to vary greatly, making this search very unstable. Consistent results were eventually achieved but we do not claim that these choices in hyperparameters were optimal, given that tuning was kept to a minimum to avoid overfitting our limited training set. As discussed in our future directions, we hope that with more data, a more robust LSTM model can be constructed for a more fair comparison to the other methods.

BERT required very minimal tuning to attain consistent and strong results. In our study, we limited the modifications of the original BERT hyperparameters to a few tweaks in the learning rate schedule. This was completed manually, on a holdout validation set (disjoint from the test set). The selected settings for the learning rates for each separate MISL macrostructure element are provided in the submitted code, the link to which is found under the *Supplementary Code* section. As with LSTMs, more tuning could have been performed, and we make no guarantees of optimality. However, as discussed in the results, the performance of these BERT models on the validation set far exceeded any of the other methods. This alone was used as justification in the acceptance of the final hyperparameter settings.

#### Quadratic Weighted Kappa

Evaluating performance of the various ML models required selection of a performance metric. While simple accuracy does provide some insight into how well these models classified the narratives to MISL scores, they do not factor in the degree of mistakes made. In other words, scoring a narrative as a 3 when it is actually a 0, is just as incorrect as scoring it as a 1 when using accuracy. Quadratic Weighted Kappa (QWK) is another performance metric that directly addresses this issue and is therefore widely used in the AES literature [34, 35]. It is calculated by taking the inner product of the confusion matrix, of predicted and actual scores, with a weight matrix where there are 0’s on the diagonal and the squared difference of predicted and actuals on the off diagonals. This is divided by the inner product of the expected value and the same weight matrix as before. This fraction of inner products is subtracted from 1, giving a single value constrained between 0 and 1. A “1” would indicate perfect agreement between raters, or in our case between the model and human scorers, and a 0 would be no agreement. An accepted baseline in the literature for strong agreement is 0.6, though we would like to see QWK between our models and a human on par with or exceeding human to human QWK.

To train our models to optimize QWK, we adapted each method to perform regression, which is equivalent to minimizing the mean squared error between predicted and actual results. This has the effect of penalizing larger misclassifications in our predictions more than smaller errors, but unfortunately outputs a real valued number between 0 and 3, instead of a discrete integer value from 0 to 3. For simplicity, we rounded our regression results from each method to the nearest integer and, while we found strong results using this approach, an argument could be made for using a smoothed form of QWK as the loss function.

## Results

The QWK for each ML model compared to an US, on either a holdout test set (TS) (80% training, 20% test set) or through 10-fold cross-validation (CV), is shown in Table 2. Of the first 3 methods (CMRF, TIRF, GVEL), none exceeded the accepted standard of agreement with the undergraduate scorers (0.60 or above) on any element of the MISL tool. Surprisingly, BERT dramatically surpassed the performance of the other methods, with all QWK’s greater than 0.9 except for “Consequence”, which still achieved a respectable 0.790. This is very strong agreement by any AES standard and shows that it is possible to construct a ML model that can consistently match the scoring ability of a trained undergraduate scorer on the MISL tool.

**Table 2:** QWK of machine learning models trained on undergraduate scored data.

MISL Element	CMRF w/ CV	TIRF w/ CV	GVEL w/ CV	BERT w/ TS
*Character*	0.504	0.595	0.317	0.975
*Setting*	0.239	0.348	0.459	0.911
*Initiating Event*	0.498	0.533	0.485	0.945
*Plan*	0.423	0.536	0.335	0.953
*Action*	0.466	0.503	0.522	0.942
*Consequence*	0.494	0.500	0.493	0.790
*Elaborated Noun Phrase*	0.480	0.437	0.454	0.908

QWK of the various ML models to the undergraduate scorers either through 10-fold cross-validation (CV) or a holdout test set (TS) as compute time permitted.

To compare these results with human-to-human QWK, we compared the BERT to undergraduate scorer results to the QWK of undergraduate scorers to an expert scorer on 50 randomly selected narratives of the same prompt. We also compared BERT to expert scores to see if we get results comparable to the undergraduate to expert QWK. From Table 3, we can see that BERT achieving high agreement to undergraduate scorers resulted in a similar pattern of agreement to the expert scores, when compared to undergraduate to expert. The relatively low QWK between undergraduate and expert scores reiterates the need for a consistent scoring system, as this is indicative of rater error in the US. It also confirms that to create an effective and reliable scoring system, it would be beneficial to train BERT on only ES data. Given enough data (≈ 400 narratives as with the US data), we expect that we can achieve similarly high QWK from BERT to expert as we achieved from BERT to undergraduates. It may even require less data than with the undergraduate scorers data as we’d expect the expert scores to be more consistent (i.e. higher signal to noise ratio).

**Table 3:** QWK of BERT to US, BERT to ES, and US to ES.

MISL Element	BERT to US	BERT to ES	US to ES
*Character*	0.975	0.938	0.956
*Setting*	0.911	0.591	0.601
*Initiating Event*	0.945	0.593	0.547
*Plan*	0.953	0.427	0.400
*Action*	0.942	0.396	0.417
*Consequence*	0.790	0.651	0.410
*Elaborated Noun Phrase*	0.908	0.724	0.780

Results of comparing the QWK of BERT to undergraduate scores (US) to that of BERT to expert scores (ES) and US to ES.

## Discussion

In this study, we aimed to investigate if machine learning methods could accurately score narrative macrostructure elements, as defined by the MISL tool, as well as if an automated narrative scoring system could generate scores at or exceeding the reliability level of human scorers. BERT was the most successful method for calculating each macrostructure element, given the constraints of our dataset, with all QWKs well above 0.6 and all but one above 0.9. A possible explanation for the success of BERT is that it is pre-trained on a very large corpus, affording it a great deal of familiarity with the English language, a technique known as transfer learning. This approach also allows the model to learn each word embedding in the context of its sequence, potentially clarifying semantically ambiguous words. Finally, BERT as well as GVEL’s usage of word embeddings allow them to handle unseen words in training, since the word embeddings form a semantically related lower dimensional space, in which unseen words can still be represented given their relationships to known words.

Alternatively, CMRF and TIRF methods were both forced to model patterns in the narratives to score these various elements from the ground up with no pre-training. For example, to score “Initiating Event”, CMRF and TIRF had to learn all of the possible ways an initiating event could be stated from only a small sample of 400 narratives, while not having any structures to handle the sequential nature of text. In practice, these models are even more limited in that they have no way to handle words that were not seen in the original training set, which could have also contributed to their poor performance on the unseen test set. These constraints severely hinder the ability of both CMRF and TIRF to handle such complex textual relationships.

GVEL on the other hand utilizes some principles of transfer learning and, through the LSTM, natively handles sequences. Given the success of LSTM’s in the literature, an LSTM trained with sufficient data could possibly perform comparably to BERT. However, with only 400 narratives to learn these types of patterns and dependencies, training an LSTM from random parameter weights was shown to be very difficult and ultimately, in our experiments, not feasible.

As the quantity of data increases though, we hope to return to LSTM’s as an alternative to BERT for a few reasons. Most importantly, while they are still not quite at the level of classical statistical learning methods (e.g. regression, decision trees, etc.) for interpretability, the literature on interpreting the results on LSTM’s is much more rich than the recently introduced Transformer models, such as BERT. While interpretation of predictions does not limit the usability of these models in a clinical setting, it may be of academic interest to understand the relationship between the ML based predictions and the rubric upon which it is based, the MISL. Also, Transformer models are notoriously large computationally with BERT-base (the model used in this paper), weighing in at 110M parameters. The scale of these models not only hinders their interpretability, but also makes them intractable for use in resource constrained environments. Two recent works [36][37] propose methods for distilling the knowledge–for lack of a better term–of BERT models down to much smaller neural networks, often LSTMs. This is a promising research direction and one we hope to explore in the future.

As stated, BERT achieved reliability levels with undergraduate scorers that were well above an acceptable threshold, as well as at or above the reliability levels of undergraduates to an expert scorer, as shown in Table 3. This confirmed that, for our specific prompt and population, it was possible to train a machine learning model to score narratives in a fashion that was on par with trained human raters.

### Rater error

As can be seen from Table 3, the QWK between undergraduate and expert scores on certain elements, particularly elements related to the initiating event (i.e. plan, consequence and action), were quite low with plan reaching a QWK of 0.400, action 0.417 and consequence 0.410. The low QWK seen between undergraduate and expert scores can likely be attributed to sources of rater error, such as rater drift and/or fatigue. Rater drift is a common issue in hand-scoring whereby the scoring “style”, or a scorer’s understanding of a concept, shifts overtime, thus causing their scoring patterns to become more lenient or severe [38]. Fatigue is another common issue, where scorers make errors in scoring simply due to becoming tired and losing focus [9]. Both of these issues likely contributed to the lower than expected agreement found between scorers, but this is precisely what will be remedied by training BERT on expert scored narratives, as it won’t be susceptible to these types of rater errors. A similar pattern was observed between expert and BERT scores.

### Clinical implications

The results of this study show great promise for the automation of narrative macrostructure scoring. While BERT will require some additional training with a greater number of expert scores to increase the scoring reliability, our results have shown that BERT can score aspects of narratives accurately with acceptable levels of reliability. These scores are consistent with those of human raters, particularly when those raters are highly experienced in using the rubric. Automating the scoring of important aspects of narratives enables more professionals to utilize narrative analysis in their respective settings. While educators, psychologists and clinicians will still at this point be required to transcribe their audio recordings, no coding of transcripts will be necessary, which will add additional time savings. Given that clinicians’ most commonly reported barrier to conducting language sample analyses was time, we feel that this technology may be helpful in overcoming that obstacle. BERT may help to breach a research-to-practice gap by allowing more consistent, reliable and authentic monitoring of narrative discourse development for student whose goals include this important milestone. Moving forward, it is our intention to make this technology open-access to the public, through the use of a simple web based applet. This applet will allow users to upload narrative transcripts and obtain automated MISL scores for various aspects of macrostructure and microstructure.

### Limitations

Given that BERT was trained exclusively on narratives elicited from the *Alien Story* picture prompt, there is currently a limitation in the generalizability of the scoring. The accuracy of MISL scores produced by BERT for different prompts is not yet known, but likely lower than narratives elicited in the same context. In addition, BERT was trained on a corpus of children aged 5-9, which may affect its generalizability to other age groups. Training BERT on a corpus of older children will be necessary to ensure that BERT can accurately predict scores for children above the age of 9.

### Future directions

While the question of how BERT made its correct (or incorrect) predictions was not answered in this work, we feel that this work provides evidence that ML is a viable alternative to human based holistic scoring of narratives, given the constraints of our data. In future work, we would like to train BERT exclusively on narratives scored by expert scorers. As we saw, BERT effectively replicated the scoring of non-expert (trained undergraduate) scorers, though this had the effect of replicating their mistakes as well. We believe that training a BERT based model on a comparable amount of narratives scored by experts would give a more reliable and consistent scoring experience to clinicians and users of the MISL rubric.

Also, as discussed in the limitations, we would like to extend our study to other narrative prompts. It is not yet known whether a single model can handle the scoring of MISL macrostructure elements across multiple prompts or if multiple distinct models will be needed. This will almost certainly impact the clinical practicality of our approach and is therefore a priority in our research.

Finally, we believe a more detailed error analysis is warranted to explore the mistakes made by our BERT based model. While the mistakes were minimal, the edge cases may provide a deeper insight into the patterns in narratives most relevant to scoring. In parallel, this research will also naturally explore the question of interpreting results from a large Transformer model. The landscape of research in this field is expanding quickly, but the context of our work may provide a shortcut to understanding, given that the scores in our data are based off of a rubric that is theoretically grounded on a well-established theory of narrative structure [16]. In the future we hope to leverage this feature of our data to contribute in the understanding of how these “black box” models understand language.

## Supplementary code

The code for the final successful BERT model can be found here: https://github.com/sharadkj/BERT_QWK_MISL/blob/master/BERT_QWK.ipynb. Instruction for running the code are in the notebook.
